# “You Know You Are Sick, Why Do You Carry A Pregnancy Again?” Applying the Socio-Ecological Model to Understand Barriers to PMTCT Service Utilization in Western Kenya

**DOI:** 10.4172/2155-6113.1000467

**Published:** 2015-06-05

**Authors:** Maricianah Onono, Zachary Kwena, Janet Turan, Elizabeth A Bukusi, Craig R Cohen, Glenda E Gray

**Affiliations:** 1 Centre for Microbiology Research, Kenya Medical Research Institute, Kisumu, Kenya; 2 Department of Health Care Organization and Policy, School of Public Health, University of Alabama at Birmingham, USA; 3 Department of Obstetrics, Gynecology & Reproductive Sciences, University of California, San Francisco, USA; 4 Perinatal HIV Research Unit, Faculty of Health Sciences, University of Witwatersrand, South Africa

**Keywords:** PMTCT cascade, HIV/AIDS, Pregnant women, Social-ecological model, Sub-Saharan Africa

## Abstract

**Objective:**

Throughout most of sub-Saharan Africa (SSA), prevention of mother-to-child transmission (PMTCT) services are readily available. However, PMTCT programs in SSA have had suboptimal performance compared to other regions of the world. The main objective of this study is to explore the socio-ecological and individual factors influencing the utilization of PMTCT services among HIV-positive pregnant women in western Kenya using a social ecological model as our analytical lens.

**Methods:**

Data were collected using in-depth interviews with 33 HIV-infected women attending government health facilities in rural western Kenya. Women with HIV-infected infants aged between 6 weeks to 6 months with a definitive diagnosis of HIV in the infant, as well as those with an HIV-negative test result in the infant were interviewed between November 2012 and June 2013. Coding and analysis of the transcripts followed grounded theory tenets. Coding reports were discussed in a series of meetings held among the authors. We then employed constant comparative analysis to discover dominant individual, family, society and structural determinants of PMTCT use.

**Results:**

Barriers to women’s utilization of PMTCT services fell within the broad constructs of the socio-ecological model of individual, family, society and structural determinants. Several themes cut across the different steps of PMTCT cascade and relate to different constructs of the socio-ecological model. These themes include: self-motivation, confidence and resilience, family support, absence or reduced stigma, right provider attitude and quality of health services provided. We also found out that these factors ensured enhanced maternal health and HIV negative children.

**Conclusion:**

The findings of this study suggest that a woman’s social environment is an important determinant of MTCT. PMTCT Interventions must comprehensively address multiple factors across the different ecological levels. More research is however required for the development of multi-component interventions that combine strategies at different ecological levels.

## Introduction

Sub-Saharan Africa (SSA) has the highest estimated numbers of pregnant women living with HIV [1,2]. When properly implemented, prevention of mother-to-child transmission (PMTCT) of HIV can prevent up to 98% of mother-to-child transmissions [3]. Although PMTCT utilization in SSA has significantly increased over the past decade, it is still far from universal. Only about 56% of women in SSA access PMTCT services [4]. In addition these programs have had suboptimal performance compared to other regions of the world [5]. In developed countries, PMTCT utilization has been shown to reduce vertical transmission to less than 2% [3] compared to 45% in SSA.

**Figure 1:**
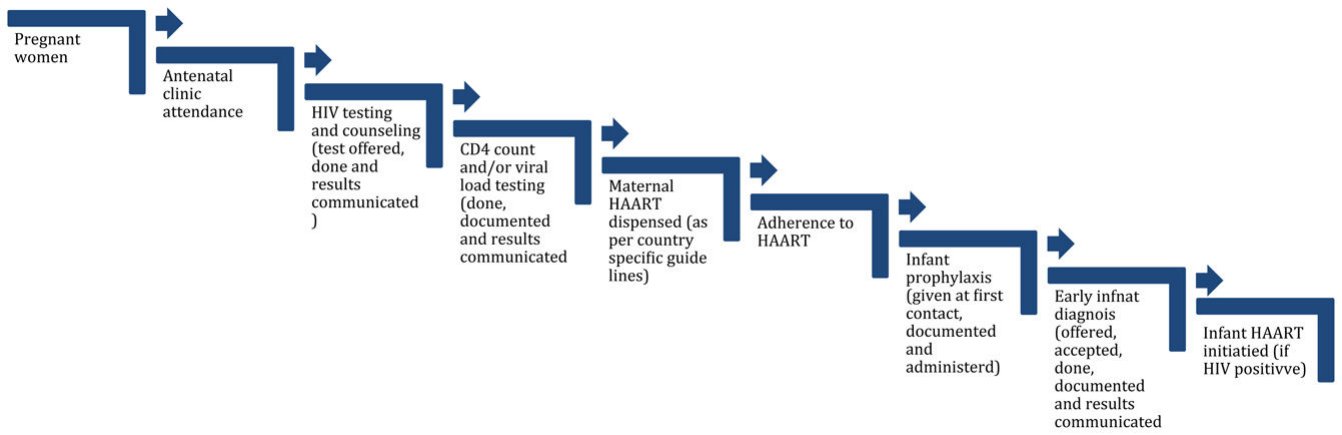
PMTCT cascade.

The PMTCT cascade describes a series of steps that HIV-infected pregnant women have to navigate to prevent HIV transmission during pregnancy, labour, delivery and breastfeeding [6,7] (Figure 1). Numerous factors affect the optimal utilization of PMTCT services by pregnant women in SSA [8] and lead to drop-off along the PMTCT cascade. These factors exist at different levels that require in-depth understanding to inform the design of programs that can promote PMTCT to eliminate paediatric HIV in SSA.

Kenya, like most countries in SSA, has experienced challenges with trying to reduce attrition from the PMTCT cascade [9]. Studies on PMTCT in Kenya have broadly assessed program effectiveness by evaluating PMTCT performance against numerical targets. Many of these studies have focused on single factors that are thought to affect uptake of PMTCT at a specific stage of the cascade [10–13]. These researches have often neglected the interface between HIV-infected pregnant women and their peers, communities and the health system and her consequent health-related behaviour. The quantitative nature of these studies often fails to capture in-depth information that can be obtained by addressing cascade-wide and context-specific factors that can inform efforts to enhance adherence to PMTCT cascade. Understanding the failures across the entire spectrum of the cascade and how an individual woman’s circumstances at each step of the cascade influences her behaviour at later stages is critically important to improve outcomes. One way to achieve this is to use an analytical framework that looks at how individuals are influenced by different circumstances of their immediate environment.

**Figure 2:**
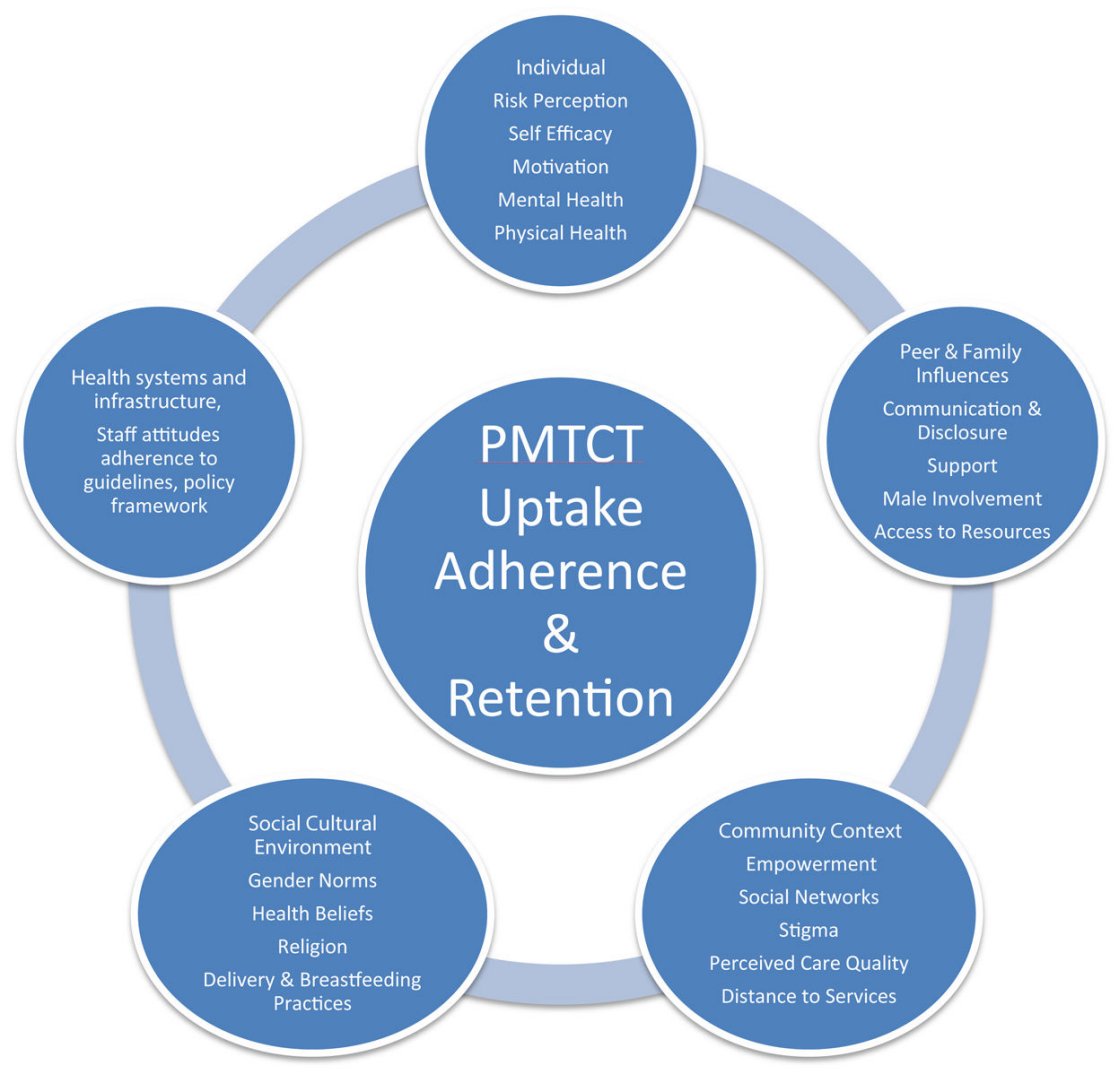
Socio-ecological model.

The social ecological model recognizes that whereas individuals are responsible for instituting and maintaining lifestyle changes necessary to reduce risk and improve health, individual behaviour is influenced by factors at different levels within their social environment [8,14] as illustrated in Figure 2. Thus, using the social ecological model as our analytical lens, the main aim of this study was to explore HIV-infected pregnant women’s interactions with their social environment in the utilization of PMTCT services in western Kenya.

## Methods

### Study design

This was a qualitative investigation conducted as part of a mixed method case control study that sought to identify factors associated with PMTCT failure in an area of Kenya with widely available free PMTCT health services [15]. The flexibility of individual in-depth interviews as qualitative methodologies enabled us to evoke meaningful and culturally salient responses pertaining to PMTCT service utilization and uptake [16]. We defined facilitators of PMTCT service utilization as any bio-psycho-social or structural factors at the individual, peer, communities or health system level that made it easier for women to utilize PMTCT services. Cases were defined as HIV-infected mothers of infants aged 6 weeks to 6 months with a definitive diagnosis of HIV in the infant. Controls were defined as HIV-infected mothers of infants aged 6 weeks to 6 months with an HIV-negative test result in the infant. Women were enrolled, as infant HIV diagnosis became known between November 2012 and June 2013.

### Study setting

The study was conducted in Nyanza in western Kenya which has the highest prevalence of HIV among adults (15.1%) [17,18]. HIV prevalence amongst pregnant women is up to 20.7%. [6]. All women were enrolled from government health facilities that are supported by the President’s Emergency Plan for AIDS Relief (PEPFAR)-funded Family AIDS Care & Education Services (FACES) program [10].

### Sampling and sample size

We conducted 33 in-depth interviews with both cases and controls. Our sample size was determined by saturation point. In-depth interview participants who were purposefully selected were a subset of the case control pairs of women participating in face-to-face structured interviews in the parent study. Informed consent was obtained prior to initiating any study activities.

### Development of interview guide

**Figure 3:**
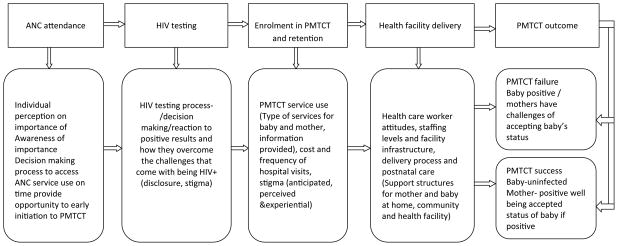
Conceptual model to describe service use and barriers over the PMTCT cascade

The in-depth interview guides used were developed in English and translated into the local language, Dholuo. The guide was developed based on social ecological theory. Under this theoretical framework, individual women’s attributes (personal and behavioural) as well as attributes of the social environment interact to produce an outcome [19]. Based on this conceptualization (illustrated in Figure 3), we developed a standardized interview guide with open-ended questions that guided the interviews. During the interview, we covered a wide range of issues that included decisions around enrolment into antenatal care, facilitators and barriers, HIV testing at ANC, reaction to test results and disclosure, enrolment into PMTCT program, adherence to doctor’s advice and medication, labour and delivery and postnatal care.

### Data collection

A trained and experienced qualitative research assistant interviewed consenting participants. The interview took about 90 minutes. The interviews were recorded by voice-activated digital recorder and later uploaded into password-protected folders on the study computer. The resultant audio-files were transcribed verbatim into Dholuo and later translated into English. The national ethical review committee for the Kenya Medical Research Institute (KEMRI) approved the study for implementation.

### Data analysis

Analysis of the transcripts took as a thematic approach based on the social ecological theoretical model, followed by a grounded approach to discover other emerging themes and sub-themes during the process of transcripts reading, exploration and coding responses [16,20]. Using Vivo 10 (QSR International Pty Ltd, Melbourne, Australia) a qualitative data analysis software, we coded the transcripts, categorizing the data into broad codes (themes) and in each identified fine codes (subthemes). Coding reports were discussed in a series of meetings held among the authors of this paper to refine the coding framework. We then employed constant comparative analysis to identify dominant structural and individual determinants of PMTCT use [21]. We further conducted sub-group analyses examining differences between women who had HIV-infected children and women whose children were not infected to identify underlying contextual factors.

## Results

### Socio-demographic description of participants

Participants had an average age of 27.7 years with two thirds (69.7%) having primary level education. The women had an average parity of 3.3. Approximately 94% of the women had disclosed their HIV status to their partners and 54% to their mothers. Ninety-four per cent of the women reported that they had attended ANC in their last pregnancy, but only 61% had received antiretroviral therapy (ART) during their pregnancy (Table 1).

### Individual level factors

There seemed to be complex set of mechanisms for decision-making and motivation to initiate antenatal care (ANC), accept the HIV test and continue with care at the facility. For most women, the decision to attend ANC was informed by the desire to know about their baby’s development, to know their HIV status or to fulfil what they perceived as a mandatory requirement by the health facilities.

“We go to the clinic when we are pregnant so that we can know if we are HIV+ or how the baby is laying in the womb” Mother aged 28 with HIV infected child

“It’s a requirement that once you find out you are pregnant you come to the clinic get tested and if found with the virus start taking medication to help prevent the child from contracting the virus and also to get vaccinated” Mother aged 29 with HIV uninfected child

“…I got advice from people who had started coming to the clinic who told me now that I was pregnant it was advisable for me to go to the clinic to know my status which could be positive or negative, and that delivering at the traditional midwife would be risky…” Mother aged 28 with HIV infected child

While some women readily accepted the HIV test and the resultant HIV positive diagnosis, majority were shocked and did not know how to go about the process of disclosure

“… when I learnt about my status I was shocked then. I could not think straight and that is why I asked them to leave me for some time I go home. Now when I went home I could not come because my husband did not know that I had tested positive. I was thinking of how I should let him know of his status too.” Mother aged 31 with HIV infected child

We observed a tendency for women with infected children to portray a lack of self-efficacy, manifesting in a delay in utilizing PMTCT services. On the contrary women with uninfected children showed a purposeful determination.

“If I had started [PMTCT] earlier I would have prevented it [baby sero-converting] but since I started late it was not possible.” Mother aged 29 with HIV infected child

“I decided not to be more depressed since when I looked around me I realized there were many people who were on drugs. So I decided to be on drugs to help me continue supporting my children.” Mother aged 34 with HIV uninfected child

Due to the same determination and resilience, women with uninfected children also seemed to find better compromises.

“Sometimes it is hectic when you are working because you have to ask for permission. Maybe if you stay at home and you are not working it’s ok, but if you are working like me then you will find that it is so difficult…coming from work, you carry the child, dress him, and come with him, so it becomes stressful at times and you must just be determined” Mother aged 33 with HIV uninfected child

### Family level factors

Majority of the pregnant women, who were diagnosed with HIV for the first time, struggled with the fear of having discordant results from that of their spouses and the consequences that may be associated with it such as violence and separation.

“Some men will become abusive and can even chase you away that; you know where you got the disease from and the woman will go away.” Mother aged 27 with HIV infected child

Some of the women perceived that such negative behaviour was acceptable.

“He has the right to chase you because he doesn’t know where you contracted the disease and you might infect him. The drunken ones would come back and shout telling you to go back to your home because you will infect them and this the information widely spreads in the community.” Mother aged 31 with HIV infected child

During disclosure women received mixed reactions from their spouses that included violence, rejection and denial that made them withdraw from services. Others, however, encountered positive and supportive environment as illustrated below

“It was easy for me, because my husband gave me support, he paid for my fare of two hundred [Kenyan shillings] to and another two hundred from the hospital and as well as fifty shillings for a drink. He also kept on reminding me about my appointments, and whenever he was away I could borrow money and he would refund when he came back” Mother aged 31 with HIV infected child

Women who already knew their HIV status tended to report experiencing lack of support from close family members for the incident pregnancy as one woman narrated below

“Like me my grandmother told me, “You know you are sick, why do you carry a pregnancy again?” “Instead of bringing up these three children of yours, do you bring up a child to suffer with the disease?” Those things. So it is very rare for them to accept you when you are pregnant” Mother aged 33 with HIV uninfected child

### Community level factors

At the community level, women expressed fear of being identified as being HIV positive. For instance, one participant said that: “If they [pregnant women] know their status they may be told to be on drugs and this brings shame to many people. Others collect their drugs from other clinics far away where they even use bus-fare to reach just to avoid the shame of other people knowing they are on HIV medication.” Mother aged 34 with HIV uninfected child. Women explained that the frequency of visits one makes to a health facility might make the community label them as HIV-positive. As such there was fear and shame of attending the ANC too frequently which some were able to overcome.

“At first when you go for clinic visits you meet a lot of people. Some of these people may start gossiping that you are sick. Now I was scared of that at first but not anymore.” Mother aged 24 with HIV infected child

“…It doesn’t scare me at all. Even if someone sees me at the hospital picking the drugs, I am no longer ashamed.” Mother aged 34 with HIV uninfected child

### Institutional level factors

Negative attitudes by health care providers, long distance to health facilities coupled with lack of money for transport and even user fee charges at the health facilities limited the number of ANC visits women make to the health facilities.”… there used to be a sister [nurse] who immediately you come to the hospital it’s like you had already annoyed her. So I don’t know because that can also make people not to come because during those days people never used to go to that hospital” Mother aged 24 with HIV infected child

Women also complained about staff neglect, harassment and complacency particularly to do with health facility delivery. The challenges were cited with the same emphasis by both mothers with HIV infected and uninfected children. For instance, one woman recalled how she was sent away when in labour. “When I went to the hospital I met a certain lady who was so harsh to me. When I told her it was time she chased me away and told me it was not yet. When I was out on the veranda I felt the baby coming and I delivered my baby. That is why am saying that here at the hospital they don’t treat us well.” Mother aged 24 with HIV infected child.

Women also reported congestion at health facilities and laxity in offering services as illustrated in the quote below: “…they delayed and also I lost a lot of blood. [They] delay in cutting the umbilical cord and losing a lot of blood. I could not walk thereafter and I also experienced blackouts” Mother aged 31 with HIV infected child.

Where health care workers were supportive and positive, they played a key role in assisting the women to disclose their HIV status in a safe environment. “If you are found to be positive you are told to bring your partner and when you come they start the process a fresh. So it looks like you are receiving your results for the first time then they now know how to handle your spouse” Mother aged 28 with HIV uninfected child

Women with uninfected children generally reported they received adequate information on what to do to prevent perinatal transmission. “They taught me and also gave me a drug which I would give the child in case I gave birth at home or on the way to hospital. So I carried the drug with me during the labour pain. On the day of delivery after he was born I told the doctor that I had the drug they had given me. The doctor took it and gave the child” Mother aged 30 with HIV uninfected child.

On the other hand women with HIV infected children reported that the information they were given at the health facility was insufficient or unclear or that they were not given anti-retrovirals as required despite having consistently attended antenatal clinics as required or advised properly about breastfeeding

“…I heard them say that there is a drug given during labour pain then after giving birth they separate your umbilical cord fast. For me this did not happen. I was not given the drug during contraction and they delayed cutting the umbilical cord. That is maybe how it happened that he got infected.” Mother aged 31 with HIV infected child.

“I was not told whether I should stop breastfeeding after six months or continue…it [information] wasn’t [sufficient] because I don’t know whether to continue breastfeeding after six months or not.” Mother aged 23 with HIV infected child.

## Discussion

The findings of this study reveal deep-seated factors for PMTCT failure which point to women’s social environment rather than the women as individuals. The main strength of this study is its unique rich qualitative data on the facilitators of PMTCT utilization from the accounts of women with both positive and negative PMTCT outcome for infants that are not found in the previous studies [8,22]. We found that barriers and facilitators to women’s utilization of PMTCT services along the PMTCT cascade fall within the constructs of the socio-ecological model. Several themes cut across the different stages of the PMTCT cascade. These themes include: self-motivation, confidence and resilience, peer/family support, absence/reduced of stigma and right provider attitude. We also found out that these factors ensured enhanced maternal health and HIV negative children. On the other hand, barriers identified include: fear of HIV status disclosure, lack of family support, enacted stigma, lack of correct information on PMTCT in the community, geographical spread health facilities in terms so distance and cost and health staff mistreatment.

Self-motivation and self-efficacy came out as strong facilitators of PMTCT utilization across the care continuum. Studies have documented the contribution of self-motivation and efficacy on the uptake of PMTCT services such as care seeking and appropriate infant feeding practices [23–25]. Unless women believe that their ANC attendance, HIV testing, enrolment into PMTCT and health facility delivery can result in their improved health and HIV negative baby, they may not have incentive to enrol and remain in PMTCT programs.

Women’s support from family and peers was a facilitator for PMTCT utilization. The importance of male involvement in the success of PMTCT program cannot be overemphasized [26,27]. A study by Aluisio et al. [27] showed that in cases when men were involved in antenatal care attendance of their spouses, there was 48% reduced chance of HIV transmission to the new born child and 45% reduced mortality of the new born children. Lack of affirmation and constructive feedback from family members, appears to affect women’s ability to uptake and adhere to PMTCT intervention guidelines. Several studies support the fact that undermining pregnant women socially may hinder their utilization of PMTCT [28,29] and result in HIV prevention failure [2,30]. A relevant example on how peer support can enhance service utilization is Mothers2mothers (m2m). M2M employs HIV-positive mothers as peer educators and care providers in clinical health facilities to provide education and psychosocial support to HIV positive pregnant women and new mothers [31–33]

Absence of stigma and discrimination came out as a strong facilitator of the uptake of PMTCT services across most levels of the cascade [12,22,34–36]. Stigma hinders HIV testing uptake, the entry point to PMTCT [10,11] and, when testing occurs stigma reduces their inclination to disclose. Women who do not disclose their HIV status are less likely to have support from their families and peers [37]. Based on HIV-positive women’s cited perceptions of fear around disclosure, addressing stigma in communities, rather than focusing on individual-level approaches, may be a more effective means of increasing PMTCT [38].

Health facility factors were identified as having influence on PMTCT uptake. Specifically, health staff attitudes and poor communication were identified as major barriers to women’s enrolment and completion of PMTCT cascade. Other studies have also had similar findings [39–41]. A multisite study in Cameroon, Cote d’Ivoire, South Africa and Zambia found that the overall quality of services offered at the health facility was important for increased PMTCT program coverage and retention of women into the program [39]. Poor quality of services offered at health facilities, including bad staff attitude and communication kept away many pregnant women from attending antenatal care clinics and consequently missing a chance to benefit from PMTCT. For instance, a study by Theuring et al. showed that service provider attitudes were a significant barrier to male involvement in antenatal care of their wives [41]. HIV-infected pregnant women are already experiencing great psychological distress and as such, healthcare providers need to use an approach that is friendly to encourage these women to enrol and remain in PMTCT program [28]. Similar to several other studies, distance to the health facilities, cost of transport and availability of means of transport came out clearly as barriers to PMTCT uptake [42–44]. Low or lack of income by many rural families coupled with poor road networks and scarcity of means of transport combine to make it difficult for many women to access antenatal care and by extension enrolment into PMTCT programs. Identifying these health facility and infrastructural barriers that also contribute significantly to women’s uptake and retention in care gives a break to over focus on individual women in these low resource settings that are historically understaffed and under financed.

There were a few limitations to this study. Due to the time that had elapsed since the antenatal period and time of HIV-positive diagnosis, women may not have been able to accurately recall information. To minimize this, we limited the timing of study recruitment to between 6 weeks to 6 months after the birth. Additionally, while the results from this study are likely to be representative of the situation in Western Kenya, it is unclear how generalizable they will be to other regions of Kenya or sub-Saharan Africa. Nonetheless, combined with information about the study setting, the results will be useful to policy makers and program planners throughout the region as they develop services to improve the PMTCT services. The main strength of our study is that we compared reports from mothers with HIV-infected infants and those with negative infants thus allowing us to reveal potential factors related to PMTCT failure at multiple levels.

## Conclusion

Apart from women’s own attributes, their social environments also play a pivotal role in HIV infected women’s use of PMTCT services. Women reporting adverse social environments tended to have HIV-infected infants as opposed to their counterparts who had supportive social environments. This implies that program planners need to use a socio ecological lens to identify previously unidentified barriers and as a guide in selecting and implementing context specific interventions. Our study findings suggest that communities, more than the individual women themselves need to bear the responsibility of PMTCT. Sustainable strategies to prevent MTCT may need to comprehensively address multitude of factors within the socio-ecological model. More research is however required to explore complex interventions combining multiple strategies across different ecological levels.

## Figures and Tables

**Table 1:** Socio-Demographic Characteristics of Participants by HIV Status of the Infant (N=33).

Attribute	Overall	HIV negative infant	HIV positive infant
	%/Mean	%/Mean	%/Mean
Mean age, years (SD)	27.7 (5.4)	28.4(5.8)	27.1(5.0)
Education level, *n (%)*			
Primary	23 (69.7)	12 (75.0)	11 (64.7)
Secondary	8 (24.2)	3 (18.8)	5 (29.4)
Other	2 (6.1)	1 (6.3)	1 (5.9)
Mean gravidity (SD)	3.2 (2.0)	3.1 (1.6)	3.3 (2.4)
Mean parity (SD)	3.3 (2.1)	3.1 (1.6)	3.4 (2.4)
HIV status disclosure, *n (%)*			
Partner	31 (93.9)	16 (100)	15 (88.2)
Mother	18 (54.5)	9 (56.2)	9 (52.9)
Everyone	2 (6.1)	2 (12.5)	0 (0)
No one	1 (3.0)	0 (0)	1 (5.9)
Other	14 (42.4)	7 (43.7)	7 (41.1)
Attended ANC in last pregnancy, *n (%)*	31 (93.9)	16 (100)	15 (88.2)
Received HAART in pregnancy, *n (%)*	20 (60.6)	11 (68.8)	9 (52.9)
Gender of children *n (%)*			
Female	17 (51.5)	9 (56.2)	8 (47.1)
Male	16 (48.5)	7 (43.8)	9 (52.9)
Place of delivery *n (%)*			
Hospital	21 (63.6)	10 (62.5)	11 (64.7)
Home	9 (27.3)	5 (31.3)	4 (23.5)
Traditional birth attendant	2 (6.1)	0 (0)	2 (11.2)
Other	1 (3.0)	1 (6.3)	0 (0)
